# Antitussive Efficacy and Safety Profile of Ethyl Acetate Fraction of *Terminalia chebula*

**DOI:** 10.1155/2013/256934

**Published:** 2013-08-19

**Authors:** Rizwan ul Haq, Abdul Wahab, Khurshed Ayub, Khalid Mehmood, M. Azhar Sherkheli, Rafeeq Alam Khan, Mohsin Raza

**Affiliations:** ^1^Department of Pharmacy, Hazara University, Havelian Campus, Havelian, Abbottabad 22500, Pakistan; ^2^Section of Pharmacology, H.E.J. Research Institute of Chemistry, University of Karachi, Karachi 75270, Pakistan; ^3^Comsats Institute of Information Technology, Abbottabad 22000, Pakistan; ^4^Department of Pharmacology, Faculty of Pharmacy, University of Karachi, Karachi 75270, Pakistan; ^5^Section of Neurosciences and Ethics, Baqiyatallah University of Medical Sciences, Tehran 19945-587, Iran

## Abstract

Antitussive effects of ethyl acetate fraction of *Terminalia chebula* on sulphur dioxide (SO_2_) gas induced cough have been examined in mice. Safety profile of *Terminalia chebula* was established by determining LD_50_ and acute neurotoxicity. The result showed that extract of *Terminalia chebula* dose dependently suppressed SO_2_ gas induced cough in mice. *Terminalia chebula*, after i.p. administration at dose level 500 mg/kg, offered maximum cough suppressive effects; that is, number of coughs at 60 min was 12 ± 1.52 (mean ± SEM) as compared to codeine 10 mg/kg; i.p., dextromethorphan 10 mg/kg; i.p., and saline, having frequency of cough 10.375 ± 0.866, 12.428 ± 0.81, and 46 ± 2.61, respectively. LD_50_ value of *Terminalia chebula* was approximately 1265 mg/kg, respectively. No sign of neural impairment was observed at antitussive doses of extract. Antitussive effect of *Terminalia chebula* was partly reversed with treatment by naloxone (3 mg/kg; s.c.) while rimcazole (3 mg/kg; s.c.) did not antagonize its cough suppression activity. This may suggest that opioid receptors partially contribute in antitussive action of *Terminalia chebula*. Along with this, the possibility of presence of single or multiple mechanisms activated by several different pharmacological actions (mainly anti-inflammatory, antioxidant, spasmolytic, antibacterial, and antiphlegmatic) could not be eliminated.

## 1. Introduction

Plants have formed the basis of sophisticated traditional medicine systems that have been in existence for thousands of years [1]. Ayurvedic, Unani, Kampo, and traditional Chinese medicine based systems continue to play an essential role in primary health care [2]. It has been estimated by World Health Organization (WHO) that approximately 80% of world's inhabitants, mainly residing in developing countries, rely on traditional medicine, and 85% of traditional medicine involves the use of plant extracts or their active principles. The plant-derived products also play a significant role in health care system of the remaining 20% population, mainly residing in developed countries. In the USA, for example, 25% of all prescriptions dispensed from community pharmacies from 1959 to 1980 contained plant extracts or active principles prepared from higher plants [3].

Most popular cough medicines throughout the world are based on herbal derivatives. The number of plants that have been accepted as antitussives by different societies is immense. For example, the USA Physician Desk Reference (PDR) for Herbal Medicine categorizes over 100 herbs as antitussives [4]. Opium, *Ammi*, coltsfoot, plantain ma huang, thyme, and so forth are used from centuries in folklore to treat coughs and other pulmonary ailments. Frequent reports have been published in the botanical and ethnopharmacological literature suggesting that traditional antitussive plants from Europe, Asia, Africa, and elsewhere may offer significant cough suppressive effects (e.g., see [5–7]). Numerous compounds such as codeine, morphine, noscapine, bromhexine, guaifenesin, ephedrine, cromolyn, and their derivatives, isolated from different plant species, are well-established western medicines for treating cough or underlying pathologies [8].

*Terminalia chebula* Retz. (family Combretaceae), locally called “Harh” or “Harir” in Urdu and Punjabi, is a tree native to subcontinent [9]. Fruits of *Terminalia chebula* are considered in ayurvedic and unani medicine as stomachic, tonic, carminative, expectorant, anthelmintics, antidysenteric, alternative, and antispasmodic [9, 10]. These are useful in asthma, bronchosis, cough, sore throat, thirst, vomiting, inflammation, tumor, bleeding piles, pains, chronic and recurrent fever, diarrhea, diabetes, dysentery, anemia, and eye diseases. Fruits, coarsely powdered and smoked in pipe, afford relief in fit of asthma [10]. *Terminalia chebula* in compound preparations offers remarkable cure for relief of phlegmatic conditions [11].

Cough can be induced in experimental animals by chemical, mechanical, or electrical stimulation of sensory nerve afferents in the larynx, trachea, or bronchial mucosa or by stimulation of CNS. However, it is considered that cough obtained by chemical stimulation is more comparable to that in humans than is obtained with other tussigenic stimuli [12]. Sulphur dioxide gas has been used to elicit cough in various experimental animals, for example, in cats [13], in rats [14], in mice [5], and so forth. In present studies, sulphur dioxide gas induced murine cough models, developed by Miyagoshi and colleagues in 1986 [5] were used to investigate antitussive efficacy of testing materials (see also [6, 7]).

## 2. Material and Method

### 2.1. Plant Material and Extraction

The dried fruits of *Terminalia chebula* were purchased from local herbal market in Karachi, Pakistan, and identified by taxonomist at the Department of Botany, University of Karachi, Pakistan. A voucher specimen has been deposited in the herbarium of Department of Botany, University of Karachi, Pakistan, for future references. The dried fruits were crushed and were soaked in ethanol for 3-4 days, and ethanol was decanted and evaporated to obtain ethanolic extract. Remaining fruit material was again extracted with ethanol in the same manner. Both extracts were mixed and then partitioned between hexane, chloroform, ethyl acetate, butanol, and water. Ethyl acetate fraction was subjected to antitussive evaluation.

### 2.2. Experimental Animals

Studies were carried out on NMRI (Naval Medical Research Institute, USA) mice weighing 20–30 g, obtained from the animal house facility of Section of Pharmacology, H.E.J. Research Institute of Chemical Sciences, University of Karachi. Animals of either sex were used. Animals were kept in groups of 5-6 in transparent plastic cages and provided standard 12 hrs light/dark cycle beginning at 8 a.m. Standard food and water were available ad libitum. With some exception, all experiments were carried out between 8 a.m. and 6 p.m. All efforts were made to minimize animals suffering and to reduce the number of animals used.

### 2.3. Chemicals

Sulphuric acid and polyethylene glycol (PEG) 400 were purchased from Sigma Chemical Company, St. Louis, MO, USA. Sodium hydrogen sulphite was purchased from Merck, Darmstadt, Germany. Codeine phosphate, dextromethorphan hydrobromide, and naloxone hydrochloride were received as gifts from Wilson Pharmaceutical Co., Islamabad, Pakistan; Abbott Laboratories (Pakistan) Limited, Karachi, Pakistan, and Haji Medicine Co., Rawalpindi, Pakistan, respectively. Rimcazole dihydrochloride was received as a gift from John Church, Department of Anatomy and Cell Biology, Faculty of Medicine, The University of British Columbia, Vancouver, Canada, and Jonathan Katz, Psychobiology Section, NIDA Intramural Research Program, National Institute of Health, Baltimore, USA.

Testing material was dissolved in PEG 400. PEG 400 was diluted in water at a ratio of 30 : 70. All the chemicals were dissolved in normal saline. All the solutions were freshly made at the day of testing and administered to a final volume of 0.1 mL/10 g body weight of mice. Testing material and all chemicals were administered intraperitoneally (i.p.), with exception of naloxone and rimcazole, which were administered subcutaneously (s.c.). All doses (of testing material and chemicals) were expressed in mg/kg of body weight of animals, excluding the weight of their salts.

### 2.4. Antitussive Evaluation

**Figure 1:**
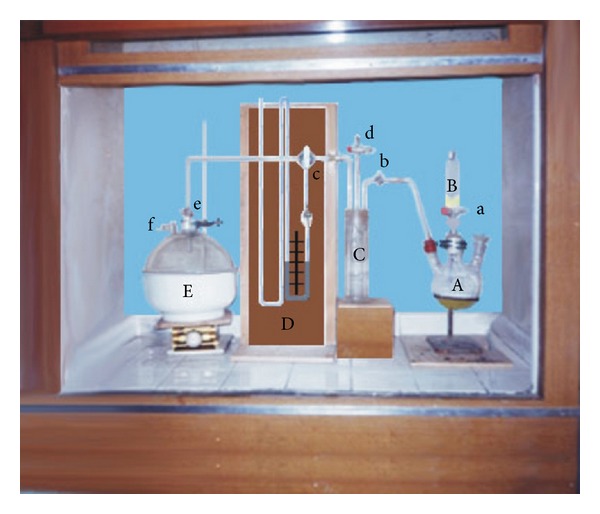
Apparatus used in sulphur dioxide gas induced cough model. A: three-necked round bottom flask, containing 39% NaHSO_3_ solution, B: dropping funnel having concentrated H_2_SO_4_, C: gas reservoir, D: water manometer, and E: desiccator. The procedure used to produce sulphur dioxide gas is described in text.

Antitussive identification and quantification of testing material were determined by sulphur dioxide (SO_2_) gas induced murine cough model described by Miyagoshi and colleagues in 1986 [5]. The experimental model is shown in Figure 1, where “A” is a 500 mL three-necked round bottom flask containing aqueous saturated (39%) sodium hydrogen sulphite solution. By opening the stopcock “a” of dropping funnel “B,” concentrated sulphuric acid (98%) was introduced to generate SO_2_ gas. SO_2_ gas filled previously in “A” and by opening stopcocks “b” and “c” pressure in gas reservoir “C” was elevated, which was recorded by the water manometer “D.” The stopcock “b” was then closed and stopcock “d” was opened slightly until the pressure in “D” reached 6 mmHg (constant throughout the experiment), then stopcock “d” was closed. These procedures were conducted in a draught. Cough response of an animal was observed by placing the animal in desiccator “E.” Stopcocks “c,” “e,” and “f” were opened, respectively, and when the pressure in “D” became 0 (zero) mmHg, all the stopcocks were closed immediately. A certain amount of SO_2_ (which was fixed throughout the experiment) was introduced into the desiccator in this way. One minute after introduction of gas, the animal was taken out of the desiccator, and number of coughs produced during the initial 5 min of exposure was counted by an up-ended filter funnel, with a stethoscope at the tip in which the mouse was confined.

Animals were divided into seven groups, containing 8 mice each. One served as control group (saline, 0.1 mL/10 g; i.p.), three groups for ethyl acetate fraction of *Terminalia chebula* EAFTc (125, 250, and 500 mg/kg; i.p.), two groups for standard drugs codeine phosphate (10 mg/kg; i.p.) and dextromethorphan (10 mg/kg; i.p.), and the remaining group was used for vehicle (PEG 400, 0.1 mL/10 g; i.p.). These doses of testing material were selected on the basis of toxicity profile and preliminary antitussive screening (data not shown). Initially, the number of coughs for each animal was determined at 0 min, before administration of any chemical or testing material. Hence, it has been illustrated that cough response to a given stimulus varies from animal to animal but that repeated assessments within the same animals are fairly reproducible [15]. Thus, animals having low or high cough threshold were not entertained for further studies. Number of coughs was observed for all animal groups at 30, 60, and 90 min (after drug administration) intervals by using same procedure. The time interval at which the greatest antitussive action was observed was taken as time of the peak effect.

### 2.5. LD_**50**_

The dose of testing material that caused death in 50% of the animals within 24 hrs (LD_50_) was calculated using the method described by Lorke in 1983 [16]. Briefly, EAFTc at the doses of 10, 100, and 1000 mg/kg was administered i.p. to groups of three mice each. Upon the results of mortality in each group after 24 hrs, 4 more mice were administered different doses of EAFTc in order to obtain the least and most toxic value, and LD_50_ was calculated by geometric mean of these values.

### 2.6. Acute Neurotoxicity

The potential of EAFTc to induce neural impairment was determined by inverted screen acute neurotoxicity test developed by Coughenour and colleagues in 1977 [17]. The apparatus consists of six 12.6 cm square platforms of 0.6 cm wire mesh supported by metal bars that in turn are mounted on a metal rod. Mice were pretested on the apparatus the day preceding the experiment, and those failing the task were not used for the subsequent drug test. For the test procedure, mice were dosed with incremental doses of EAFTc (125, 250, 500, and 1000 mg/kg; i.p.), placed 30, 60 and, 120 min on individual platforms and rod-rotated through an arc of 180°. Mice unable to climb to an upright position for 1 min duration were rated as failures [17]. Eight mice were used for each dose.

### 2.7. Elucidation of Mechanism of Action

To determine the possible mechanism of antitussive effects of *Terminalia chebula*, we used a nonselective opioid receptors antagonist naloxone to antagonize the cough suppressive activity of EAFTc at different doses. In detail, effects of naloxone (3 mg/kg, s.c.; 10 min pretreatment) were examined against the antitussive activities of saline (0.1 mL/10 g, i.p.; 30 min pretreatment), opioid receptor agonist, codeine (10 mg/kg, i.p.; 30 min pretreatment), sigma (*σ*) receptor agonist, dextromethorphan (10 mg/kg, i.p.; 30 min pretreatment), and EAFTc (250 and 500 mg/kg, i.p.; 1-hour pretreatment) [18, 19]. Pretreatment times and doses were selected for each compound based on experiment conducted in this study and published reports [18, 19]. The effects of naloxone on frequency of coughs were also determined by s.c. administration of different doses of naloxone, and results were compared with normal saline. In another set of experiments, we also tested the effects of sigma receptors antagonist rimcazole against antitussive effects of different doses of EAFTc. Briefly, effects of rimcazole (3 mg/kg, s.c.; 45 min pretreatment) were determined against saline (0.1 mL/10 g, i.p.; 30 min pretreatment), codeine (10 mg/kg, i.p.; 30 min pretreatment), dextromethorphan (10 mg/kg, i.p.; 30 min pretreatment), and EAFTc (250 and 500 mg/kg, i.p.; 1-hour pretreatment) [18, 19]. The effects of rimcazole on frequency of coughs were also determined by s.c. administration of different doses of rimcazole, and results were compared with normal saline.

### 2.8. Statistics

Data were analyzed using statistical analyzing software “Microcal Origin version 6.” The number of coughs was expressed as mean values ± SEM. Student's *t*-test was performed for each experiment to determine the difference between control and experimental groups. Difference was considered significant if *P* values were less than 0.05.

## 3. Results

### 3.1. Antitussive Efficacy

**Figure 2:**
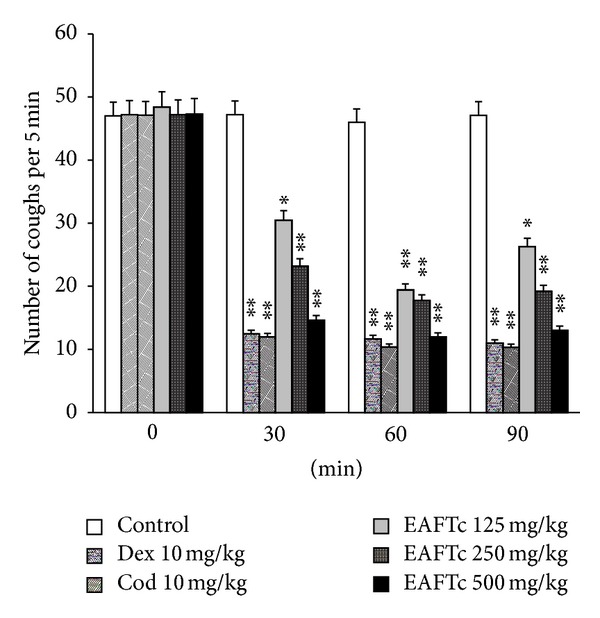
Effect of EAFTc (ethyl acetate fraction of *Terminalia chebula*) (125, 250, and 500 mg/kg) on sulphur dioxide gas induced cough by i.p. administration at different time intervals. Number of coughs were counted 5 minutes after cough challenge and expressed as mean ± SEM, obtained in 8 mice in each group. Significant differences from saline control were indicated as *P < 0.01 and **P < 0.001 by Student's *t*-test.

Intraperitoneally (i.p.) administered codeine (10 mg/kg) inhibited SO_2_ gas induced coughing in mice (Figure 2). Codeine reduced frequency of cough from 47.125 ± 2.21 to 10.375 ± 0.56 (*P* < 0.001), that is, 77.98% inhibition in incidence of coughing at 60 min interval after drug administration. Dextromethorphan (10 mg/kg; i.p.) dose-dependently inhibited cough produced by SO_2_ gas in mice (Figure 2). At 10 mg/kg cough was inhibited by 73.55% at 60 min after drug administration (*P* < 0.001). As illustrated in Figure 2, the inhibitory effects of codeine (10 mg/kg; i.p.) and dextromethorphan (10 mg/kg; i.p.) were maintained for at least 90 min (*P* < 0.001).

i.p. administration of EAFTc significantly decreased number of coughs induced by SO_2_ gas in a dose-dependent manner (*P* < 0.01; *P* < 0.001). As shown in Figure 2, EAFTc (125 and 250 mg/kg) exhibited maximum inhibition of coughing at 60 min, and then there was a slight decrease in antitussive action of EAFTc after 60 min. However, EAFTc 500 mg/kg produced long duration of action after i.p. administration with TPE 60 min, and cough inhibition was maintained up to at least 90 min. The maximum inhibition of cough produced by the extract was at 500 mg/kg dose level; incidence of coughs was decreased from 47.28 ± 1.79 (mean ± SEM) to 12 ± 1.52 (mean ± SEM) at 60 min after drug administration, whereas codeine (10 mg/kg; i.p.) exhibited a decrease in number of coughs from 46 ± 2.61 to 10.375 ± 0.565 (mean ± SEM) at the same time interval. These observations reveal that EAFTc (500 mg/kg; i.p.) was as active as codeine (10 mg/kg; i.p.) and dextromethorphan (10 mg/kg; i.p.). PEG 400 (10 mL/kg; i.p.) alone was without influence on the incidence of coughs in the given time frame after SO_2_ gas challenge; the incidence of coughs was 47.125 ± 1.301, 46.875 ± 1.563, 45.75 ± 1.578, and 46.875 ± 1.44 at 0 min (before PEG administration) and 30, 60, and 90 min after PEG administration, respectively. So it is clear that the vehicle (PEG) does not integrate in cough inhibition provoked by EAFTc.

### 3.2. Toxicity Profile

#### 3.2.1. LD_**50**_

The LD_50_ of ethyl acetate fraction of *Terminalia chebula* after i.p. dosing was calculated to be 1264.91 mg/kg per 24 hrs. Extract produced purgative effect in most of animals before death.

#### 3.2.2. Acute Neurotoxicity

The effects of EAFTc on neural impairment and motor function were determined by inverted screen acute neurotoxicity test [17]. EAFTc was observed at different doses (125, 250, 500, and 1000 mg/kg; i.p.) and time intervals (30, 60, 90, and 120 min) in mice. *Terminalia chebula* did not express any noticeable sign of acute neurotoxicity at any specified time when tested up to 1000 mg/kg.

### 3.3. Effect of Naloxone on Antitussive Activity of *Terminalia chebula*

**Figure 3:**
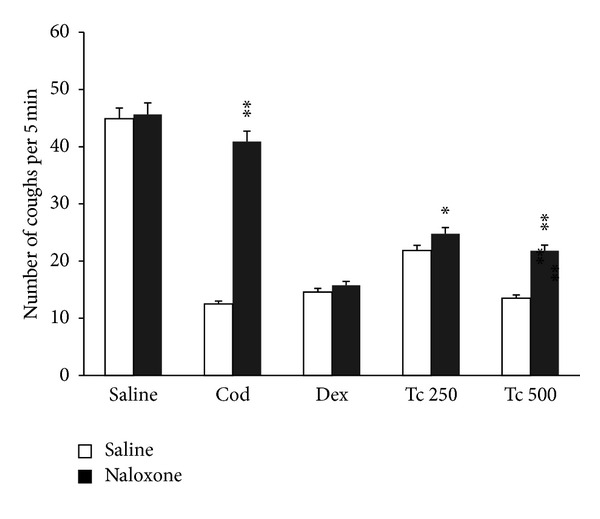
Effects of naloxone (3 mg/kg) on the antitussive effects of cod (codeine 10 mg/kg), dex (dextromethorphan 10 mg/kg), and Tc 250 (ethyl acetate fraction of *Terminalia chebula *250 mg) and Tc 500 (ethyl acetate fraction of *Terminalia chebula *500 mg). Naloxone was injected s.c. 10 min before cough challenge. Codeine and dextromethorphan were injected i.p. 30 min while testing materials were administered i.p. 60 min before cough challenge in mice. Each column represents mean with SEM for at least 8 mice. Significant differences from saline control were indicated as *P < 0.05 and **P < 0.01 by Student's *t*-test.

A nonselective opioid receptor antagonist naloxone (up to 4 mg/kg after s.c. administration) alone was without any significant influence on the incidence of cough after SO_2_ gas challenge, representing mean values of cough efforts 44.88 ± 1.46, 44 ± 1.21, and 45.62 ± 1.39(mean ± SEM) at 30 min after s.c. dosing of 1 mg/kg, 2 mg/kg, and 4 mg/kg of naloxone, respectively. Mice treated with codeine (10 mg/kg; i.p.) coughed an average of 12.5 ± 0.867 times (Figure 3), which represented 72.82% inhibition of the cough reflex. In the group of codeine-treated animals that received naloxone (3 mg/kg; s.c.), the incidence of cough was 40.875 ± 1.60 (*P* < 0.01), demonstrating an attenuation of the antitussive effect of codeine by naloxone (Figure 3). Naloxone (3 mg/kg; s.c.) did not antagonize inhibitory effects of dextromethorphan (Figure 3). Naloxone partially antagonized the inhibitory responses produced by intraperitoneally administered EAFTc (250 and 500 mg/kg), as illustrated in Figure 3 (*P* < 0.05 and *P* < 0.01, resp.).

### 3.4. Effect of Rimcazole on Antitussive Activity of *Terminalia chebula*

**Figure 4:**
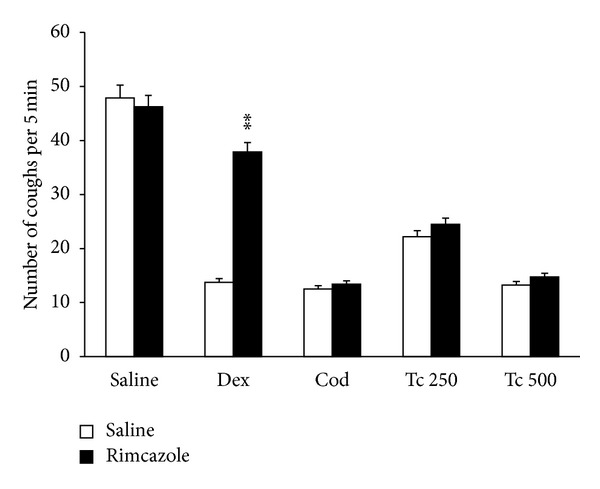
Effect of rimcazole (3 mg/kg; s.c) on the antitussive effects of dex (dextromethorphan 10 mg/kg; i.p.), codeine (10 mg/kg; i.p.), and Tc (ethyl acetate fraction of *Terminalia chebula *250 and 500 mg/kg; i.p.). Rimcazole was administered 45 min before cough challenge. Codeine and dextromethorphan were administered 30 min before cough induction, and testing material was administered 60 min before cough induction in mice. Each column represents mean with SEM for at least 8 mice. Significant differences from saline control were indicated as *P < 0.05 and **P < 0.01 by Student's *t*-test.

The *σ*-receptor antagonist rimcazole (up to 10 mg/kg; s.c.) alone had no effect on SO_2_ gas induced cough in mice, showing mean values of cough efforts 47.375 ± 1.32, 46.25 ± 1.82, and 45.25 ± 1.62 (mean ± SEM) at 30 min after s.c. dosing of 3 mg/kg, 6 mg/kg, and 10 mg/kg rimcazole, respectively. The *σ*-receptor agonist dextromethorphan (10 mg/kg; i.p.) inhibited SO_2_ gas induced coughs in mice. As illustrated in Figure 4, the antitussive activity of dextromethorphan (10 mg/kg; i.p.) was significantly inhibited by rimcazole (3 mg/kg; s.c.): cough incidence = 13.75 ± 0.881 coughs in dextromethorphan-treated animals versus 37.875 ± 1.652 coughs in the animals treated with rimcazole and dextromethorphan (*P* < 0.05). In contrast, rimcazole did not influence the antitussive activity of codeine (10 mg/kg; i.p.) and EAFTc (250 and 500 mg/kg; i.p.), as shown in Figure 4 (*P* > 0.05).

## 4. Discussion

Cough is the most common respiratory symptom that has been experienced by every human. It is an essential protective and defensive act whose action secures the removal of mucus, noxious substances, and infections from the larynx, trachea, and larger bronchi. On the other hand, a number of patients have nonproductive cough, which is not associated with mucus clearance and may have a different stimulation. It may be the first overt sign of disease of airways or lungs and may significantly contribute to the spread of airborne infections and, in some instances, may result in severe functional and structural damage to the organism. The primary action of currently available cough suppressants (opiates, dextromethorphan, etc.) is on the central cough pathway. The significant side effects of these agents such as constipation, respiratory depression, dependence, drowsiness, and death from this action limit their use in human [20]. There is a current huge unmet need for the development of safe, effective antitussive therapeutic options in the treatment of persistent cough as alternative to existing medications.

Safety is one of the major concerns in the development of new treatment for cough. Opiates with excellent cough suppressant activity offer some relief to patients with cost of numerous severe and life-complicating side effects such as the attenuation of respiratory centre activity, decrease of secretion, increase of sputum viscosity, decrease in expectoration, dependence, bronchoconstriction, and constipation [21]. Most of these adverse effects are produced as a result of their centrally mediated actions at brainstem level. Thus testing material should offer antitussive activity with minimum neurotoxicity, and antitussive doses should be lower than doses responsible for impaired motor function, characterized by ataxia, sedation, altered motor activity, and impaired righting reflex. Inverted screen acute neurotoxicity test was used to quantify the effect of testing material on motor function. In this test, compounds with sedative and/or ataxic properties produce dose-dependent increase in screen test failures, whereas other classes of drugs (e.g., psychomotor stimulants) do not. *Terminalia chebula* did not elicit motor impairment and ataxia when tested up to 1000 mg/kg. The result of acute toxicity tests indicated that after i.p. dosing EAFTc possessed a more favorable acute safety profile when compared to findings by others for currently used compounds, namely, codeine and dextromethorphan. These observations illustrate that this extract possesses wide therapeutic range.

*Terminalia chebula* seems to have a quite good ability to inhibit chemically induced cough. The ability of *Terminalia chebula* in various doses to suppress coughing was compared to commonly used drugs in clinical practice. The excellent cough suppression activity of codeine, a narcotic antitussive, is accompanied with serious side effects, which could limit its use [21]. Testing material was compared with another antitussive, dextromethorphan, which has fewer unwanted effects. The tested substance, *Terminalia chebula*, at dose level 125 mg/kg has slightly lower activity than codeine 10 mg/kg and dextromethorphan 10 mg/kg. However, at higher doses (500 mg/kg), it has antitussive activity similar to codeine (10 mg/kg) and dextromethorphan (10 mg/kg). These results provide pharmacological evidence in support of folklore claims as an antitussive agent. 

To determine whether the antitussive activity of EAFTc was attributed via activation of opioid receptors, it was tested in presence of naloxone, a nonselective opioid receptor antagonist. Codeine was used as a standard opioid receptor agonist whose antitussive activity has been shown previously to be inhibited by naloxone [22]. The antitussive activity of EAFTc at lower dose level (250 mg/kg) was slightly reversed with treatment by naloxone. However, cough suppressant activity of EAFTc at higher doses was more significantly reduced by naloxone. It seems plausible to assume that higher doses of EAFTc were required to induce opioid-mediated inhibition of sulphur dioxide gas provoked cough in mice. This data provides evidence that antitussive activity of EAFTc at low doses was not mainly mediated by opioid receptors but increasing activity of EAFTc at higher doses could be explained as a result of opioid receptor-mediated inhibition.

Dextromethorphan is a commonly used nonnarcotic antitussive, which has a greater affinity for sigma receptor [23]. In this study, reduction in antitussive action of dextromethorphan by treatment with rimcazole was confirmed [18]. Rimcazole, at the dose which significantly inhibited the antitussive activity of dextromethorphan, did not affect the antitussive activity of EAFTc. This finding suggests that antitussive effect of EAFTc in this model is independent of activity at the sigma receptor.

It is more likely to suggest that EAFTc may exert antitussive action via multiple mechanisms, both centrally and peripherally. Plant extracts exhibit multiplicity of mechanism, which is activated by several different types of chemical compounds [24]. Apart from opioid and sigma receptor agonists, recent understandings of pharmacology and physiology of cough have introduced several centrally located targets (GABA-B receptor agonists, tachykinin receptor antagonists, and nociceptin), which play a leading role in modulating cough reflex [25–27]. The possible involvement of any one or more of these in antitussive activity of EAFTc could not be excluded. 

EAFTc may possess peripheral antitussive activity via suppressing one or more components of peripheral limb of cough reflex. Phytochemicals have great ability to mask airways nerve endings (irritant receptors) due to their mucilaginous properties [28, 29]. The anti-inflammatory and antiallergic potential of *Terminalia chebula* [30] could be another factor accountable for its peripheral mechanism. Existence of opioid receptors in periphery is well recognized [22, 31]. It seems more logical that opioid-mediated cough suppression may be achieved from periphery rather than centrally. Hence, pharmacokinetic profile of EAFTc is a matter of great concern, and that will decide whether EAFTc enters CNS or does not. 

An increase in exogenous or endogenous oxidative stress can protease-antiprotease imbalance within the alveolar structures to the development of chronic inflammation [32] and emphysema [33]. This established role of oxidants in lung tissue injury and disease has promoted the hypothesis that antioxidants may act as preventive agents. Several reports conclude that a high concentration of antioxidants like ascorbic acid and alpha tocopherol could be one of the factors inhibiting chronic cough [34, 35]. The extract of fruits of *Terminalia chebula* possesses potent antioxidant activity [36–38]. Furthermore, the influence of airway muscle contractility may be another factor influencing cough, as *Terminalia chebula* possesses antispasmodic activity [30, 39, 40].

Multiplicity of constituents present in the extract may yield massive interactions. Some components may enhance the intensity of activity of active agent at opioid receptor by exerting synergistic effects. Still others may lessen the opioid-mediated antitussive efficacy of extract. The possibility of presence of more than one active principle with different site of actions could not be eliminated. Further fractionalization of the extract of *Terminalia chebula* may enhance the possibility of getting active principle(s)/fraction(s) with more precise mechanism.

It is supposed that several pharmacological properties (mainly anti-inflammatory, antioxidant, spasmolytic, antibacterial, and antiphlegmatic) may contribute in antitussive efficacy of *Terminalia chebula. *These pharmacological properties of extract of *Terminalia chebula *may validate the popular use of this herb in cough related to numerous respiratory diseases.

## 5. Conclusion

The present study indicates that extract of *Terminalia chebula *possesses antitussive activity against sulphur dioxide gas evoked cough in mice. This activity of the plant was partly reversed by naloxone, but it was not affected with treatment by rimcazole, indicating a partial involvement of opioid receptors in inhibition of coughing. In addition, the antitussive potential of this plant correlates with various pharmacological properties, which may justify its widespread use in various respiratory conditions in traditional medicine. Further studies aimed at isolation of the active compounds responsible for antitussive activity are ongoing in our laboratory.
